# Quality of Care for HIV Infection Provided by Ryan White Program-Supported versus Non-Ryan White Program-Supported Facilities

**DOI:** 10.1371/journal.pone.0003250

**Published:** 2008-09-22

**Authors:** Patrick S. Sullivan, Maxine Denniston, Eve Mokotoff, Susan Buskin, Stephanie Broyles, A. D. McNaghten

**Affiliations:** 1 Centers for Disease Control and Prevention, Atlanta, Georgia, United States of America; 2 Department of Epidemiology, Emory University, Rollins School of Public Health, Atlanta, Georgia, United States of America; 3 Michigan Department of Community Health, Detroit, Michigan, United States of America; 4 Public Health–Seattle & King County, Seattle, Washington, United States of America; 5 Louisiana Department of Public Health, New Orleans, Louisiana, United States of America; School of Public Health and Tropical Medicine, Tulane University, United States of America

## Abstract

**Background:**

The Ryan White HIV/AIDS Care Act (now the Treatment Modernization Act; Ryan White Program, or RWP) is a source of federal public funding for HIV care in the United States. The Health Services and Resources Administration requires that facilities or providers who receive RWP funds ensure that HIV health services are accessible and delivered according to established HIV-related treatment guidelines. We used data from population-based samples of persons in care for HIV infection in three states to compare the quality of HIV care in facilities supported by the RWP, with facilities not supported by the RWP.

**Methodology/Principal Findings:**

Within each area (King County in Washington State; southern Louisiana; and Michigan), a probability sample of patients receiving care for HIV infection in 1998 was drawn. Based on medical records abstraction, information was collected on prescription of antiretroviral therapy according to treatment recommendations, prescription of prophylactic therapy, and provision of recommended vaccinations and screening tests. We calculated population-level estimates of the extent to which HIV care was provided according to then-current treatment guidelines in RWP-supported and non-RWP-supported facilities. For all treatment outcomes analyzed, the compliance with care guidelines was at least as good for patients who received care at RWP-supported (vs non-RWP supported) facilities. For some outcomes in some states, delivery of recommended care was significantly more common for patients receiving care in RWP-supported facilities: for example, in Louisiana, patients receiving care in RWP-supported facilities were more likely to receive indicated prophylaxis for *Pneumocystis jirovecii* pneumonia and *Mycobacterium avium* complex, and in all three states, women receiving care in RWP-supported facilities were more likely to have received an annual Pap smear.

**Conclusions/Significance:**

The quality of HIV care provided in 1998 to patients in RWP-supported facilities was of equivalent or better quality than in non-RWP supported facilities; however, there were significant opportunities for improvement in all facility types. Data from population-based clinical outcomes surveillance data can be used as part of a broader strategy to evaluate the quality of publicly-supported HIV care.

## Introduction

Since 1990, the Federal Government, through Title XXVI of the Public Health Service (PHS) Act as currently amended by the Ryan White HIV/AIDS Treatment Modernization Act of 2006[1] (Ryan White Program, or RWP), has provided funding to states, cities, and nonprofit organizations to improve the quality and availability of medical care and supportive services for low-income, uninsured, and underinsured individuals and families affected by HIV/AIDS. Administered through the Health Resources and Services Administration (HRSA), RWP funds are provided directly to healthcare facilities (through Part C grants to Community Health Centers, University-affiliated medical centers, hospitals, or other community-based health care settings) or may support care in facilities indirectly, through grants provided to state health departments and local health departments in eligible metropolitan areas (EMAs) or transitional grant areas (TGAs).

The legislation which provides these funds for HIV care and services also requires that service providers establish quality management programs to assess the extent to which HIV health services provided to patients under the grant are consistent with the most recent PHS guidelines for the treatment of HIV/AIDS and related opportunistic infections [1]. To monitor quality of care, HRSA provides technical assistance to grant recipients for quality improvement [2], and grantees can use a proportion of their awards to implement a clinical quality management program [1].

Recently, the Institute of Medicine recommended that quality of care should be measured at the broader population level, that population-based methods should be used for such evaluations, and that information on quality of care with respect to both prophylaxis and treatment should be measured [3]. Representative data on patients in care will soon be available in the United States [4]; we used data from a pilot probability sample of patients in care for HIV infection in 1998 to provide historical information about the quality of care provided in facilities supported by the RWP to that in non-RWP-supported facilities, to provide baseline data for comparison with future analyses of quality of care from population-based systems, and to illustrate the use of population-based clinical outcomes surveillance data for describing quality of care.

## Methods

The Survey of HIV Disease and Care (SHDC) project was a pilot project to develop methods for the use of population-based sampling of persons receiving care for HIV infection as a method of HIV clinical outcomes surveillance. The methods have been previously reported [5]. The three participating health jurisdictions (“study sites”) were chosen by a competitive application process to CDC. Project staff at each study site first defined a geographic area for inclusion in the study; the geographic areas were the entire state of Michigan, health regions 1,2,3,4, and 9 in Louisiana (southern Louisiana, including New Orleans and Baton Rouge), and King County (including Seattle) in Washington State. The chosen geographic areas within each state reflected a number of considerations, including jurisdiction for public health surveillance, available resources, and distribution of AIDS prevalence within the area. Health department staff in each study site then constructed a sampling frame of HIV care facilities within the defined geographic area, using data on health care providers and facilities who had reported diagnosing or caring for persons with HIV infection to the health department as part of HIV/AIDS surveillance, and other data sources. Facilities that provided no clinical care, such as HIV counseling and testing facilities, were excluded. Facilities could represent a single provider, a group of providers sharing a common medical records system, or some other clinic with a single medical records system. Facilities were classified based on size of HIV patient load (small, medium, or large), urban vs. rural location, and on whether or not the provider or facility received RWP support–either directly from HRSA under Part C (formerly Title III), or indirectly through a state or local health department funded under Part A or B (formerly Title I or II). Receipt of RWP support was thus identified at the facility level; no determination was made at the patient level as to whether RWP resources supported specific aspects of that patient's care (such as provision of antiretroviral drugs). HIV care facilities were sampled, using probability proportional to size of the patient population, within size, urban/rural, and RWP-support strata. For this analysis, we excluded five facilities in Louisiana that provided only inpatient care, because the RWP is designed to pay for outpatient care.

From each eligible participating HIV care facility sampled, the health department requested information about the number and demographic characteristics of patients who had been seen in the facility at least once for care for HIV infection during 1998. The numbers of patients were obtained at the facility level, such that if multiple HIV clinicians were practicing in a facility that share a common medical records system, only one tally of patients would be obtained for the whole facility. Based on this information, patients were stratified within facilities on race and sex, and sampled using systematic sampling within strata from an ordered list. The sampling interval was varied in different race/sex strata to ensure adequate representation of women and racial/ethnic minorities. Of note, we did not collect information on other qualitative aspects of the facilities, such as training or experience with providing HIV care.

For each sampled patient, medical records were abstracted for the period January 1–December 31 1998. Abstractors in all study sites received the same standardized training, and used standardized definitions for clinical outcomes and laboratory measures. Data were collected on laboratory values (including CD4+ T-lymphocyte count and HIV RNA concentration [viral load]); prescription of highly active antiretroviral therapies (HAART) and prophylactic medications for the prevention of *Pneumocystis jirovecii* pneumonia (PCP) and *Mycobacterium avium* complex (MAC); provision of recommended screening tests (tuberculin skin test, Pap smear) and influenza vaccination; and information about inpatient hospital utilization. Using treatment guidelines current in 1998 [6], [7], standard definitions were constructed for which patients were eligible for recommended care (e.g., PCP prophylaxis for patients with a CD4 count <200 cells/μL). Details of the definitions for recommended care are included in Appendix S1. Quality assurance procedures (e.g., independent re-abstraction of a small sample of records and/or computerized checks that data were valid [within an expected range]) were implemented in all study areas.

Sampling weights were constructed for each patient by multiplying the sampling weight of the facility by the sampling weight of the patient within the facility. Further details of sampling weights and calculation of variance have been previously reported [5]. These weights were used to estimate the number of patients in care within the geographic areas, as well as the number of patients in care at facilities supported by HRSA and at other facilities. For each geographic area, the proportion of eligible patients receiving care according to treatment guidelines, with 95% confidence intervals, was estimated. Statistically significant differences between proportions were determined using ?^2^ tests. For some outcomes such as number of laboratory tests performed within a time period, the median number of tests per unit time, with 95% confidence intervals, was estimated; median-split ?^2^ tests were used to test for significant differences between medians. All analyses were performed in SUDAAN to account for the complex sampling design.

The SHDC project was considered to be non-research by the Centers for Disease Control and Prevention Institutional Review Board (IRB), and as such did not require IRB review. Of the three participating state and local health departments, the protocol was reviewed and received Institutional Review Board (IRB) approval in two, and in one, it was determined to be exempt from IRB review.

## Results

Overall, 95% (41/43) of eligible sampled health care facilities agreed to participate in the survey (range by site: 86%–100%). Information was abstracted from the medical records of 831 patients (range by site: 169–374); of these, 250 patients (30%) received their care in facilities supported by the RWP (range by site: 43–131: 20%–45%). Using weighted sums of patients in care, we estimated that our study made statistical inference to 18,720 patients in care for HIV infection: 8,490 (45%, CI = 29%–62%) in care at RWP-supported facilities, and 10,230 (55%, CI = 37%–71%) in care at facilities not supported by the RWP.

Limited information was collected about the 41 participating facilities. The proportions of included facilities that were RWP-supported facilities in Michigan, King County, and Southern Louisiana were 14%, 25%, and 20%, respectively. Median numbers of patients in care in the RWP-supported facilities in each of the three areas (1130, 187, 1783) were higher than the median number of patients in care in the non-RWP facilities (24, 84, 46). There was a trend for larger patients loads in RWP-supported facilities in Michigan and southern Louisiana (p = 0.06 and p = 0.07 respectively by median test), but not in King County (p = 0.52). By categorical size of patient load, in Michigan 38% of facilities had patient loads <20, 48% had patient loads 20–199, and 14% had patient loads ≥200. Corresponding proportions for Louisiana were 50%, 20%, and 30%, and for King County were 0%, 67%, and 33%.

There were some statistically significant differences in the demographic and clinical characteristics of persons receiving care in RWP-supported and non-RWP supported facilities, and these differences were not consistent in the three study areas (Table 1). Women in King County were more likely to receive care in RWP-supported facilities than in non-RWP-supported facilities, whereas the opposite was true for men. Persons aged 45 years or older in King County and Louisiana were less likely to receive care in RWP-supported facilities than in facilities not supported by the RWP, while the opposite was true for persons aged 25–44. There were racial/ethnic differences in the proportions of patients receiving care in RWP-supported and non-RWP-supported facilities in King County: 66% of patients receiving care in RWP-supported facilities were white, non-Hispanic, but 82% of patients receiving care in non-RWP-supported facilities were white, non-Hispanic. There were also differences in the distribution of risk for HIV acquisition (southern Louisiana and King County) and clinical stage of disease (southern Louisiana) between patients receiving care in RWP-supported facilities and those receiving care in non-RWP-supported facilities. In Michigan, there were no significant differences in the demographic characteristics of those receiving care in RWP-supported and non-RWP-supported facilities.

**Table 1:** Estimated characteristics of persons in care for HIV infection by Ryan White Program support status, King County, Washington, southern Louisiana and Michigan, 1998.

	King County (n = 288)		Southern Louisiana (n = 169)		Michigan (n = 374)	
	RWP supported (n = 131)	non RWP supported (n = 157)	RWP supported (n = 43)	non RWP supported (n = 126)	RWP supported (n = 76)	non RWP supported (n = 298)
Characteristic	Estimated % (SEM)	Estimated % (SEM)	Estimated % (SEM)	Estimated % (SEM)	Estimated % (SEM)	Estimated % (SEM)
**Sex**						
Male	82.1 (2.3)*	92.1 (2.8)	68.1 (3.8)	75.3 (4.0)	67.1 (8.5)	72.7 (3.3)
Female	17.9 (2.3)	7.9 (2.8)	31.9 (3.8)	24.7 (4.0)	32.9 (8.5)	27.3 (3.3)
**Age**						
13–24	1.9 (0.9)†	2.7 (1.7)	6.5 (5.1)*	9.8 (3.0)	5.1 (3.2)	3.1 (1.4)
25–44	87.8 (3.4)	63.9 (3.7)	85.2 (3.7)	59.0 (6.3)	64.0 (0.9)	71.0 (3.9)
45+	10.3 (3.3)	33.4 (4.1)	8.3 (1.4)	31.2 (5.9)	30.9 (2.8)	25.9 (2.9)
**Race**						
White, non-Hispanic	66.4 (4.5)‡	82.4 (2.7)	30.9 (14.6)	43.6 (4.8)	28.5 (8.1)	35.5 (6.6)
Black, non-Hispanic	18.1 (3.2)	10.5 (1.8)	65.3 (13.4)	48.9 (5.3)	63.1 (10.1)	59.9 (6.1)
All other/unknown	15.5 (3.1)	7.1 (1.6)	3.8 (1.2)	7.5 (3.3)	8.4 (2.0)	4.6 (1.5)
**Risk category**						
MSM	42.0 (5.6) †	58.3 (4.8)	32.1 (15.3)†	33.3 (5.8)	29.1 (9.6)	24.5 (5.2)
MSM/IDU	13.8 (4.3)	7.0 (2.9)	5.2 (0.4)	5.1 (2.6)	6.0 (5.7)	1.9 (1.4)
IDU	9.8 (2.9)	4.4 (3.1)	43.9 (16.9)	8.4 (2.3)	14.6 (4.1)	15.4 (2.4)
HRH	13.7 (2.5)	5.1 (2.0)	10.6 (1.7)	13.0 (3.2)	9.5 (6.7)	8.8 (1.3)
Other/unknown	20.7 (4.7)	25.2 (2.9)	8.2 (0.3)	40.2 (5.3)	40.8 (7.4)	49.4 (5.2)
**Clinical/immunological**						
CD4 0-99 or AIDS-defining opportunistic illness	35.7 (5.4)	41.1 (8.9)	19.7 (1.6)†	41.5 (4.5)	45.5 (5.4)	41.0 (2.0)
CD4 100-349	29.3 (5.2)	22.8 (5.7)	43.9 (2.7)	25.3 (4.5)	18.9 (5.6)	10.6 (2.4)
CD4≥350 or unknown	35.0 (5.4)	36.1 (4.1)	36.4 (4.4)	33.2 (4.7)	35.6 (9.8)	48.4 (3.1)

RWP = Ryan White Program; SEM = standard error of the mean; MSM = men who have sex with men; IDU = injecting drug user; HRH = high risk heterosexual; CD4 = CD4+ T-lymphocyte count, in cells/μL. Indicated p-values are for the overall test for difference and do not necessarily identify the specific levels of the variable that differ between RWP and non-RWP funded providers.

* p≤0.01 for ?^2^ test comparing patients at RWP and non-RWP funded providers within study site.

† p≤0.001 for ?^2^ test comparing patients at RWP and non-RWP funded providers within study site.

‡ p≤0.05 for ?^2^ test comparing patients at RWP and non-RWP funded providers within study site.

For most clinical care outcomes evaluated, there were no statistically significant differences in the quality of care provided to patients in RWP-supported and non-RWP-supported facilities (Table 2). Where statistically significant differences were observed, in each case, the proportion of patients receiving care according to treatment guidelines was higher for patients receiving HIV care in RWP-supported facilities. Patients receiving HIV care in RWP-supported facilities were more likely to receive indicated PCP or MAC prophylaxis during 1998 in southern Louisiana; were more likely to receive a tuberculin skin test during 1998 in King County; and were more likely to receive a Pap smear in 1998 in all three study areas. There were no significant differences in the median number of viral load tests, CD4 counts, or outpatient visits between patients receiving HIV care at RWP-supported versus non-RWP-supported facilities in any of the 3 study areas (Table 3). In Louisiana, patients receiving their HIV care in RWP-supported facilities were less likely to have had a hospital visit during the year than patients receiving care in non-RWP supported facilities.

**Table 2:** Estimated proportions of persons in care for HIV infection treated according to guidelines, by Ryan White Program funding status King County Washington, southern Louisiana and Michigan, 1998.

	Patients receiving treatment					
	King County n = 288		Southern Louisiana n = 169		Michigan n = 374	
	RWP-supported n = 131	not RWP-supported n = 157	RWP-supported n = 43	not RWP-supported n = 126	RWP-supported n = 76	not RWP-supported n = 298
Care provided	Treated/eligible* Estimated % (95% CI)	Treated/eligible Estimated % (95% CI)	Treated/eligible Estimated % (95% CI)	Treated/eligible Estimated % (95% CI)	Treated/eligible Estimated % (95% CI)	Treated/eligible Estimated % (95% CI)
HAART†	76/111 66% (55, 77)	90/131 69% (58, 80)	22/34 61% (56, 66)	70/98 71% (57, 82)	36/55 74% (47, 100)	125/194 65% (55, 75)
PCP prophylaxis‡	56/66 85% (74, 96)	66/83 76% (52, 100)	13/15 86% (66, 95)§	44/67 60% (47, 73)	28/32 87% (85, 89)	116/142 84% (76, 93)
MAC prophylaxis∥	14/21 71% (47, 95)	14/29 60% (40, 81)	4/5 87% (41, 98)‡‡	12/28 43% (22, 66)	9/13 81% (73, 89)	27/64 45% (14, 76)
TB test**	98/130 79% (70, 88)¶	66/157 44% (28, 61)	39/43 88% (24, 99)	51/126 42% (32, 52)	26/76 39% (0, 87)	103/297 30% (14, 47)
Influenza vaccine	36/131 31% (21, 41)	46/157 25% (15, 35)	13/43 28% (26, 29)	50/126 36% (24, 50)	21/76 38% (0, 86)	29/298 7% (2, 12)
Pap smear††	37/62 61% (49, 72)§	21/47 40% (24, 55)	12/15 81% (42, 96)‡‡	12/34 41% (24, 60)	13/22 62% (32, 92)‡‡	20/84 19% (5, 32)
Any Viral load assay	119/131 91% (85, 98)	150/157 96% (93, 100)	39/43 88% (24, 99)	96/126 71% (60, 80)	46/76 73% (37, 100)	155/298 51% (28, 74)
Any CD4 measurement	120/131 92% (86, 98)	151/157 97% (93, 100)	39/43 88% (24, 99)	102/126 80% (72, 87)	48/76 77% (46, 100)	190/298 61% (49, 72)

RWP: Ryan White Program; HAART: Highly active antiretroviral therapy; PCP: *Pneumocystis jirovecii* pneumonia; MAC: *Mycobacterium avium* complex; TB: tuberculosis; CD4: CD4+ T-lymphocyte count in cells/μL; CI: confidence interval.

* Treated/eligible = number of persons receiving care/number of persons eligible for care

† Eligibility for HAART was defined as CD4<500, PCR>20000, bDNA>10000 or AIDS-defining opportunistic illness diagnosis (any time before or during 1998)

‡ Eligibility for PCP prophylaxis was defined as CD4<200 or PCP diagnosis (any time before or during 1998)

§ p≤.001 for ?^2^ comparing percentages of patients at RWP and non-RWP funded providers within study site

∥ Eligibility for MAC prophylaxis was defined as CD4<50 cells/μL or *M. avium* complex diagnosis (any tine before or during 1998)

‡‡ p≤.05 for ?^2^ comparing percentages of patients at RWP and non-RWP funded providers within study site

** Eligibility for TB test was defined as follows: no tuberculosis diagnosis or sputum culture positive to *M. tuberculosis* prior to 1998.

¶ p≤.01for ?^2^ comparing percentages of patients at RWP and non-RWP funded providers within study site

†† The only exclusions from this analysis were men, and women with a diagnosis of invasive cervical cancer and age 13–17 with non-sexual transmission mode.

**Table 3:** Estimated health care utilization of persons in care for HIV infection, by Ryan White Program funding status, King County Washington, southern Louisiana and Michigan, 1998.

	Patients receiving treatment					
	King County n = 288		Southern Louisiana n = 169		Michigan n = 374	
	RWP-supported n = 131	not RWP-supported n = 157	RWP-supported n = 43	not RWP-supported n = 126	RWP-supported n = 76	not RWP-supported n = 298
Utilization measure	Estimated Median # (95% CI) or % (SEM)	Estimated Median # (95% CI) or % (SEM)	Estimated Median # (95% CI) or % (SEM)	Estimated Median # (95% CI) or % (SEM)	Estimated Median # (95% CI) or % (SEM)	Estimated Median # (95% CI) or % (SEM)
Viral loads*	1.0 (0.8, 1.5)	1.3 (1.0, 1.4)	1.1 (0.5, 1.5)	1.1 (0.6, 1.6)	0.5 (0.0, 1.5)	0.1 (0.0, 1.4)
CD4 counts*	1.0 (0.8, 1.5)	1.2 (1.0, 1.4)	1.1 (0.5, 1.5)	1.1 (0.9, 1.6)	0.6 (0.0, 1.5)	0.6 (0.0, 1.0)
Outpatient visits†	5.1 (4.1, 5.9)	6.4 (5.7, 8.4)	4.3 (3.3, 6.6)	5.3 (3.6, 7.6)	6.4 (2.9, 12.1)	2.2 (0.0, 4.5)
Hospital visits†						
None	85% (3.6)	85% (9.5)	91% (4.7)‡	65% (6.2)	17% (11.1)	10% (4.9)
One	9% (2.7)	10% (6.0)	7% (3.5)	24% (5.1)	53% (11.4)	61% (7.1)
Two or more	6% (2.5)	5% (3.7)	2% (1.2)	11% (4.0)	30% (10.9)	29% (5.0)
Hospital days§						
1–2	12% (7.0)	45% (13.4)	NC∥	NC∥	14% (5.9)	16% (3.7)
3–7	46% (13.0)	27% (16.0)	NC∥	NC∥	40% (10.7)	33% (4.3)
8 or more	42% (12.1)	28% (6.8)	NC∥	NC∥	46% (13.2)	51% (3.0)

RWP: Ryan White Program; SEM: standard error of the mean; CD4: CD4+ T-lymphocyte.

* Number of tests per 6 months

† Number of visits in study year

‡ p≤.01 for ?^2^ comparing percentages of patients at RWP and non-RWP funded providers within study site. The p-value is for the overall test for difference and does not necessarily identify the specific levels of the variable that differ between RWP and non-RWP funded providers.

§ Number of days for those who were hospitalized during the study year. The number of patients in each calculation is: King County, RWP = 23, non-RWP = 16; MI, RWP = 37, non-RWP = 181.

∥ NC: Not calculated. For Louisiana, RWP and non-RWP funded providers are not compared on the number of hospital days due to the very small number of patients (n = 3) at RWP supported providers who had been hospitalized during the study year.

## Discussion

We used data from a population-based sample of patients receiving HIV care in these three geographic areas to describe the quality of HIV care delivered in 1998 in RWP-supported and non-RWP-supported facilities. We observed that patients receiving HIV care at facilities supported thorough RWP funds, administered directly or indirectly through HRSA, received care which was in compliance with then-current treatment guidelines at least as often as patients who received care from non-RWP supported facilities. We believe the recommended standards of care we evaluated represent important and objective measures of quality of care, and are in alignment with HRSA's currently-proposed clinical performance measures [8]. We therefore conclude that care in RWP-supported facilities was at least of equivalent quality to care supported by other payers – and in some cases, of higher quality.

The primary strength of our study is that the patients included were selected using probability sampling methods, and are therefore representative of all patients in care for HIV infection in the three participating geographic areas. However, our study also had some weaknesses. In one site, two eligible sampled facilities refused participation, which, to the extent that the refusing facilities provided a different quality of care from participating facilities, could introduce some bias to our findings. In this case, none of the refusing facilities in the sample of facilities was RWP-supported. The King County site had no small facilities in their sample. As well, our data are somewhat dated, although we believe that the data are appropriate for documenting baseline measures of quality of care by RWP status, and demonstrating how the Institute of Medicine's recommendation to use population-based data to evaluate quality of care can be operationalized using data from a population-based, clinical outcomes surveillance project.

Also, data were only collected about care reflected in the medical records of the facility where the patient was sampled. Therefore, for patients who received HIV care in multiple facilities, the extent to which indicated care was received may have been underestimated. If patients receiving care in non-RWP-supported facilities were more likely than those receiving care in RWP-supported facilities to receive certain services, such as tuberculin skin tests or Pap smears, outside of the facility where they were sampled, then our observed differences in the proportions of patients receiving recommended screening tests may be due to misclassification. Certain of our data, such as the low estimate of receipt of viral load tests in Michigan, suggest that the extent of incomplete data due to this limitation may be pronounced for some variables in some project sites. This concern may be especially relevant to the provision of certain services, that are more likely to be provided by specialists (e.g., Pap tests) or may be more accessible and less expensive outside of HIV care facilities (e.g., influenza vaccine). Our analysis of data from women with HIV infection from a different study indicated that women who received their gynecological care at the same clinic as their HIV care were more likely to receive Pap tests as recommended [9].

The designation of RWP-support is somewhat artificial, in that RWP support was only identified at the facility level. In reality, some patients may receive support for HIV care which is received in a facility not directly supported by the RWP. For example, patients receiving care from private facilities may receive funding for purchasing medicines through the AIDS Drug Assistance Program (ADAP). Thus, our data may underestimate the extent of RWP support for care, but unless receiving ADAP impacted the care outcomes we analyzed, this should not represent a source of misclassification bias with respect to our primary conclusions.

In some cases, our precision was low and our statistical power to detect actual differences may have been limited, even when point estimates appeared very different. This occurred for two reasons. For some clinical care outcomes, our survey had large design effects [5]; for example, the design effect in the Michigan sample for influenza vaccination was 29.6. In other cases, for example for MAC prophylaxis in Michigan, there were relatively small numbers of patients for whom the clinical service was indicated; this also led to broad confidence intervals and nonsignificant ?^2^ tests.

HRSA has long-standing quality of care standards, provides technical assistance to grantees for evaluating quality of care, and has supported independent evaluations of quality of care in RWP-supported facilities [2]. Our analysis complements those previous efforts in several ways. For example, data from the HIV Costs and Services Utilization Study (HCSUS), a nationally representative sample of patients in care for HIV infection [10], was evaluated to describe how patients receiving care at RWP-supported facilities were different from those receiving care in other facilities and evaluated differences in the types of services provided at clinics [11]. They found that patients in RWP-supported facilities in 1996–1997 were more likely to be younger, less educated, poorer, female, non-white, and uninsured. We found similar results with respect to sex, age, and race in some or all of our sites for care received in 1998. The HCSUS analysis reported that RWP-supported clinics provided more types of support services than other clinics[11], but did not report individual level care outcomes, as we do in our analysis. Other evaluations have also addressed programmatic issues of service provision at the facility level, but not at the client level [12].

HRSA has recently taken steps towards development of a client-level reporting system to capture information on care at the client level, including supporting pilot activities [13]. However, comparable data from a representative sample of non-RWP-supported facilities will remain an important point of comparison when client-level data are reported directly to HRSA in the future.

Other reports have evaluated quality of care within RWP-supported facilities, but did not have a comparative group of non-RWP facilities in the same analysis. For example, Wilson et al conducted an in-depth analysis of quality of care within 68 RWP-supported facilities, and reported on similar care outcomes as do we, but further stratified their analyses by provider type. A separate analysis on the same data suggested that, because clinical care outcome measures were not highly correlated within facilities, multiple care outcomes should be evaluated [14]. We evaluated five of the outcome measures that were identified in that previous work (HAART prescription, PCP prophylaxis, tuberculosis screening, cervical cancer screening, and influenza vaccination) as well as MAC prophylaxis and measures of frequency of CD4 count and HIV viral load tests.

We report data from a public health surveillance project, not from a health services research study. It is important to recognize that the view of care offered by a clinical outcomes surveillance system is necessarily and appropriately different from the view that may be offered by health services research studies. A research study to describe the drivers of compliance with care guidelines may consider factors such as patient load, number of infectious disease specialists, sex of provider, and provider training [15]–[17]. Clinical outcomes surveillance data, on the other hand, seek to describe differences when they occur–a high level view of quality and opportunities for improvement in care, regardless of their underlying drivers. To the extent that differences are suggested by surveillance data, more in-depth evaluations and research studies may be needed to suggest steps for quality improvement. The Institute of Medicine report explicitly recognized that, despite the fact that measures of quality derived from population-based systems reflect “the cumulative effects of many influences”, population-based measures are “essential in monitoring HIV care … and identifying areas for improvement” [3]. These data were designed to be interpreted for this purpose within the local sites; therefore we did not combine data across sites in this analysis.

Although we set out to determine whether differences in quality of care existed for patients in RWP-supported facilities, it is equally important to recognize that there were important opportunities for improving quality of care across the practice settings that we included in our analysis. For example, the proportions of patients receiving indicated TB screening, influenza vaccination, PCP prophylaxis, and Pap screening were all relatively low. Separate analyses of patient-level factors associated with lack of receipt of TB screening [18], influenza vaccination [19] and PCP prophylaxis [20] have been recently published from other US clinical outcomes surveillance data. As well, it has been recently suggested that gaps in recommended HIV care in the United States may sometimes result from prioritization of limited resources by health care providers [14]. Again, more specific research is needed to determine whether observed gaps in compliance with treatment guidelines should be addressed with more training, better systems to track delivery of needed care and identify needs at the client level, more resources for care provision, or a combination of these interventions. Additionally, new models for care delivery should be evaluated for their ability to improve delivery of indicated clinical preventive services [21].

The experiences of this pilot project have been used to inform the development of a nationwide surveillance system of HIV clinical outcomes and health care, called the Medical Monitoring Project (MMP) [4]. MMP is a national probability sample of patients in care for HIV infection, constructed by multi-stage probability sampling including a probability proportional to size (PPS) sample of states, a PPS sample of facilities within selected states, and an equal probability sample of patients within selected facilities [22]. The sampling strategy in MMP is based on the methods used in SHDC, but aims to improve these by using equal probability sampling methods (EPSEM). Based on the findings from this analysis that the numbers of persons in care at RWP-supported facilities are considerable, we have also decided that stratification of the sampling frame of facilities on the basis of receipt of RWP funding is not necessary to obtain a sufficient number of patients receiving care in RWP-supported facilities in our sample. Therefore, stratification of the facility sampling frame by RWP support status will not be included in future sampling designs.

As part of future chart abstractions, in addition to documenting whether selected facilities were RWP-supported, abstractors will attempt to document RWP-supported care or services, even when they are not received in a RWP-supported facility (e.g., ADAP funding), in order to better document the impact of the provision of RWP funds to support HIV care and services (information on ADAP support of an individual patient's care would only be informative for receipt of medications, such as PCP or MAC prophylaxis, or HAART). In addition, MMP will ascertain all HIV-related care received by abstracting medical records at all facilities at which HIV-related care was received for each enrolled patient. Data from the first nationally-representative sample from MMP will be publicly available in 2009.

Data from clinical outcomes surveillance projects are primarily used for resource planning, allocation, and prioritization in state and local health departments [23]. This analysis demonstrates that data already collected as part of ongoing surveillance efforts may also be useful for evaluation of quality of care on a population level. As HRSA moves to develop client-level data systems to better document the provision of care and services supported by RWP funds [13], CDC will continue to ensure that population-based clinical outcomes surveillance data collected by state and local health departments are measured in ways that are consistent with publicly available standards for clinical performance outcomes developed by HRSA. By so doing, CDC will collect comparable data from non-RWP supported facilities that can provide a context in which to interpret the quality of care evaluations conducted by HRSA and its grantees.

## Supporting Information

Appendix S1 (0.03 MB DOC)
