# Supplementary material for: Quality of Care for HIV Infection Provided by Ryan White Program-Supported versus Non-Ryan White Program-Supported Facilities
Source: PLoS One. 2008 Sep 22;3(9):e3250. doi: 10.1371/journal.pone.0003250 (PMC2535568; doi:10.1371/journal.pone.0003250)
Supplement: Appendix S1 — (0.03 MB DOC) [file pone.0003250.s001.doc]

**Appendix: Definitions of standards of care**

Highly Active Antiretroviral Therapy (HAART):

**Met standard:** Prescription of AZT+(ddI, ddC, or 3TC)+(any protease inhibitor or any non-nucleoside reverse transcriptase inhibitor), D4T+(ddI or 3TC)+ (any protease inhibitor or any non-nucleoside reverse transcriptase inhibitor), Protease inhibitors included saquinavir, ritonavir, indinavir, and nelfinavir. Non-nucleoside reverse transcriptase inhibitors included delavirdine and nevirapine.[6]

**Eligibility:** History of an AIDS-OI diagnosis, CD4 <500, PCR > 20000, or bDNA >10000.[6]

*Pneumocystis carinii* (now *jirovecii*) pneumonia (PCP) prophylaxis:

**Met Standard**: Prescription of dapsone, pentamidine, or trimethoprim-sulfamethoxazole.

**Eligibility:** History of PCP diagnosis or CD4 <200
*Mycobacterium avium* complex (MAC) Prophylaxis

**Met Standard:** Prescription of clarithromycin, azithromycin, or rifabutin.

**Eligibility:** History of disseminated MAC or CD4 < 50 cells/µL

Tuberculosis skin test (TST)

**Met standard:** Any tuberculin skin test during 1998.

**Eligibility:** No tuberculosis diagnosis or sputum culture positive to *M. tuberculosis* prior to 1998.

Influenza vaccine

**Met Standard:** Received influenza vaccination during 1998

**Eligibility:** All persons observed
